# Minimizing Pulse Check Duration Through Educational Video Review

**DOI:** 10.5811/westjem.2020.8.47876

**Published:** 2020-10-20

**Authors:** David Yamane, Patrick McCarville, Natalie Sullivan, Evan Kuhl, Carolyn Robin Lanam, Christopher Payette, Anahita Rahimi-Saber, Jennifer Rabjohns, Andrew D. Sparks, Keith Boniface, Aaran Drake

**Affiliations:** * George Washington University, Department of Emergency Medicine, Washington DC; † George Washington University, Department of Anesthesiology and Critical Care Medicine, Washington DC

## Abstract

**Introduction:**

The American Heart Association Guidelines for Cardiopulmonary Resuscitation (CPR) recommend pulse checks of less than 10 seconds. We assessed the effect of video review-based educational feedback on pulse check duration with and without point-of-care ultrasound (POCUS).

**Methods:**

Cameras recorded cases of CPR in the emergency department (ED). Investigators reviewed resuscitation videos for ultrasound use during pulse check, pulse check duration, and compression-fraction ratio. Investigators reviewed health records for patient outcomes. Providers received written feedback regarding pulse check duration and compression-fraction ratio. Researchers reviewed selected videos in multidisciplinary grand round presentations, with research team members facilitating discussion. These presentations highlighted strategies that include the following: limit on pulse check duration; emphasis on compressions; and use of “record, then review” method for pulse checks with POCUS. The primary endpoint was pulse check duration with and without POCUS.

**Results:**

Over 19 months, investigators reviewed 70 resuscitations with a total of 325 pulse checks. The mean pulse check duration was 11.5 ± 8.8 seconds (n = 224) and 13.8 ± 8.6 seconds (n = 101) without and with POCUS, respectively. POCUS pulse checks were significantly longer than those without POCUS (P = 0.001). Mean pulse check duration per three-month block decreased statistically significantly from study onset to the final study period (from 17.2 to 10 seconds [P<0.0001]) overall; decreased from 16.6 to 10.5 seconds (P<0.0001) without POCUS; and with POCUS from 19.8 to 9.88 seconds (P<0.0001) with POCUS. Pulse check times decreased significantly over the study period of educational interventions. The strongest effect size was found in POCUS pulse check duration (P = −0.3640, P = 0.002).

**Conclusion:**

Consistent with previous studies, POCUS prolonged pulse checks. Educational interventions were associated with significantly decreased overall pulse-check duration, with an enhanced effect on pulse checks involving POCUS. Performance feedback and video review-based education can improve CPR by increasing chest compression-fraction ratio.

## INTRODUCTION

### Background

Cardiopulmonary resuscitation (CPR) in the emergency department (ED) is a multidisciplinary effort to save a patient’s life through return of spontaneous circulation (ROSC). Minute changes in CPR quality, such as the percentage of hands-on time, correlate with survival.^1^ The American Heart Association recommends that pulse checks last a maximum of 10 seconds and that the ratio of time spent performing compressions to the total duration of CPR be 80% or higher, as these correlate with increased ROSC and survival to hospital discharge.^2^ Prior studies found improved survival in patients with cardiac arrest due to ventricular fibrillation with chest compression fraction (CCF) of 0.6–0.8 and improved ROSC in patients with cardiac arrest without ventricular fibrillation with a CCF of 0.8–1.0.^3^, ^4^ In 2005 Valenzuela et al found that “frequent interruption of chest compressions results in no circulatory support during more than half of resuscitation efforts.” Since then, many other studies have emphasized the importance of CCF and its relationship to outcomes including likelihood of ROSC and survival.^3^–^5^ A recent study identified the importance of teamwork and communication as contributory factors to effective CPR.^7^ Post-arrest debriefing as a means of quality improvement has not been shown to be a positive effect.^8^

Prior studies have identified video review as one method toward improving both the technical and interpersonal aspects of CPR.^9^ Providers use video review of high-fidelity simulation training to improve skills and identify human factors associated with performance.^7^,^10^,^11^ Video review of simulations is an effective means of teaching team competencies as well as technical skills.^12^ Early use of clinical videorecording involved mostly surgical and anesthesia specialists, where researchers and at times groups of providers in conference reviewed analog video.^13^,^14^ Since then, video review has become standard practice at many trauma centers to analyze behavior and improve treatment.^13^,^15^,^16^

Investigators have shown associations between the use of point-of-care ultrasound (POCUS) and prolongation of hands-on time during arrest.^17^ Multiple studies have demonstrated the utility of POCUS to help determine the cause of a cardiopulmonary arrest, direct resuscitation efforts, assist procedures, and identify patients for whom continued resuscitative efforts would be futile.^18^–^20^ The opportunity to glean potentially management-changing information has led to widespread use of POCUS during CPR, especially in academic ED settings.^21^ However, using POCUS to assess cardiac activity may reduce “hands-on” time during resuscitation.^17^,^19^ These findings raise concerns that POCUS may inhibit effective CPR and negatively impact patient outcomes.

### Importance

Despite multiple studies showing the benefit and impact of POCUS during CPR, uncertainty exists about the potential for patient harm due to increased pulse check durations.^17^,^21^,^22^ We explore ways to minimize time spent on pulse checks in which ultrasound is used, and maximizing the CCF. Furthermore, post-resuscitation recollections of events during CPR are often inaccurate.^23^ Video review circumvents poor provider recall and offers an opportunity for quantitative data analysis of resuscitations including pulse check duration.^24^ Through the introduction of improved methods of ultrasound use and video-based feedback we may improve CPR and outcomes.

**Population Health Research Capsule**

What do we already know about this issue?

*Pulse checks under 10 seconds improve outcomes in cardiopulmonary resuscitation (CPR). Use of point-of -care ultrasound (POCUS) during CPR lengthens pulse check duration*.

What was the research question?

Does CPR video review with feedback and education improve pulse check times with POCUS use?

What was the major finding of the study?

*Educational intervention with video review was associated with reductions in pulse check duration*.

How does this improve population health?

*Adoption of an educational protocol that incorporates video review may lead to improved CPR pulse check durations and potentially patient outcomes in cardiac arrest*.

### Goals of this Investigation

Using multidisciplinary grand rounds educational sessions and individualized objective feedback, we sought to reduce pulse check duration, both with and without POCUS.

## METHODS

### Study Design and Setting

This was a prospective cohort study evaluating the use of ultrasound during CPR between December 2017–July 2019 in the ED of a single urban, academic hospital with an emergency medicine residency. The study conforms to the Strengthening the Reporting of Observational Studies in Epidemiology (STROBE) guidelines and was approved by our institutional review board (IRB# 031819).^25^

A videorecording system in three resuscitation bays continuously collected audio and video for review. Triage providers placed patients presenting to the ED with out-of-hospital cardiac arrest (OHCA) in these videorecorded bays if they were available at the time of the patient’s arrival or if nursing staff was able to move patients based on prehospital notification. Investigators collected data by reviewing the video footage and corresponding medical records. They collected data points in accordance with the Cardiac Arrest Registry to Enhance Survival (CARES).^26^ The principal investigator (DY) trained junior researchers on performing video review for two weeks. The research team met on a monthly basis to evaluate videos, and a subset (50) of videos underwent review by two study researchers to assess for interobserver variability. For each case, at least one reviewer was a postgraduate year (PGY) -3 or -4 resident and the second reviewer was a postgraduate from any year (1–4).

### Selection of Participants

The educational intervention included all ED practitioners, including attending physicians, residents, advanced providers, nurses, and technicians, who cared for adult patients presenting to the ED after OHCA who were placed in one of the three resuscitation areas with videorecording capability. All resuscitation teams included at least one attending and one PGY-2, -3, or -4 resident physician. Each team included a minimum of three nurses for documentation, medication administration, and bedside care. All staff members who participated in resuscitations were already being videorecorded per existing departmental policy. We consented willing staff members through an electronic opt-out method that included background information about the study, the subjects’ role in the study, and their ability to opt out without risk of harm or reprisal. No staff members chose to opt out of this study.

Inclusion criteria were patients older than 18 years with OHCA who were transported to our urban, tertiary care hospital. Exclusion criteria included patients suffering traumatic arrest, death pronounced prior to arrival, ROSC prior to arrival with pulse on arrival to the ED, resuscitation in a room without video capabilities, or failed video capture. Investigators did not collect data on cardiac arrest patients who were not placed in a videorecorded room. The number of available videorecorded arrests that met inclusion and exclusion criteria during the study period determined our sample size.

### Interventions

After review of a case, the reviewing team sent individualized feedback over Health Insurance Portability and Accountability Act-secure email to all involved care providers (technicians, nurses, resident physicians, and attending physicians). Providers received quantitative measures of performance including time to intravenous access, time to monitor, pulse check duration, and CCF. These summaries also included subjective feedback on ways to improve these quality metrics.

Bi-monthly presentations occurred during protected emergency medicine (EM) educational time attended by resident physicians, attending physicians, advanced practitioners, nurses, and EM technicians. A study representative (PGY-3 or PGY-4 EM resident) presented selected cases with a complete review of video footage, followed by a lecture on relevant topics related to CPR. Lecture topics included the following: team roles; POCUS; treatment of persistent ventricular fibrillation; airway management during CPR, team communication; post-resuscitation care; the presence of family during resuscitation; use of recombinant tissue plasminogen activator in cardiac arrest; the Lazarus phenomenon; CPR-induced consciousness; and termination of resuscitation. During review of the video, the presenter paused at specific times to highlight teachable moments. The attending principal investigator reviewed these prior to each presentation and the team designed teaching moments to highlight opportunities for improvement.

Presenters emphasized limiting hands-off time and shortening pulse checks. They shared POCUS-specific strategies to shorten pulse checks including positioning the probe in the desired location prior to pauses for pulse checks, counting the seconds aloud during image acquisition, and recording images during the pulse check for interpretation after CPR was resumed. We recommended a “pulse check ready” list prior to pausing CPR, including placing fingers on the pulse, ensuring that the monitor was in sight line of the resuscitation leader, and that the ultrasound probe was placed on the patient prior to the pulse check.

### Measurements

Arrival time was the time of transition from the emergency medical services (EMS) stretcher to hospital gurney. Study data included all pauses in compressions, including pauses for procedures, pulse checks, compression device malfunction, or other causes. Once providers achieved ROSC, investigators considered the case complete. Investigators calculated time of death as the time providers announced the death to the room. Time of ROSC was the time a palpable pulse was announced by either the resuscitation leader or provider who palpated the pulse. Data extracted from the audiovisual record or the electronic health record included the use of ultrasound during each pulse check, the time of each pulse check, and the ultimate outcome of the patient. Only the clinical team made the decisions of when and whether to use POCUS during pulse checks. Investigators did not include final pulse checks (during which ROSC was achieved or the resuscitation efforts were terminated). The type of compressions provided, either automated device or manual, was recorded.

Multiple team members reviewed a subset of video recordings (50) to analyze interobserver variability. Discordantly recorded times of pulse checks were averaged. Investigators did not include pulse checks recorded by one reviewer and omitted by second reviewer nor pulse checks in which reviewers disagreed on whether or not ultrasound was used. While performing data collection, the reviewers independently assessed each video and were blinded to each other’s recorded values.

### Outcome

The primary outcome was pulse check duration with and without the use of POCUS.

### Analysis

We performed univariate analyses of pulse checks with the Mann-Whitney U test and Spearman’s rank correlation coefficient, ρ, to evaluate pulse check length trends overall, with ultrasound use, and without ultrasound use. Interrater reliability between reviewers of pulse check lengths was analyzed by way of intraclass correlation coefficient (ICC) and Pearson’s correlation coefficient, r. All statistical analysis was performed using SAS version 9.4 (SAS Institute Inc., Cary, NC) with *P* < 0.05 considered statistically significant.

## RESULTS

**Figure 1:**
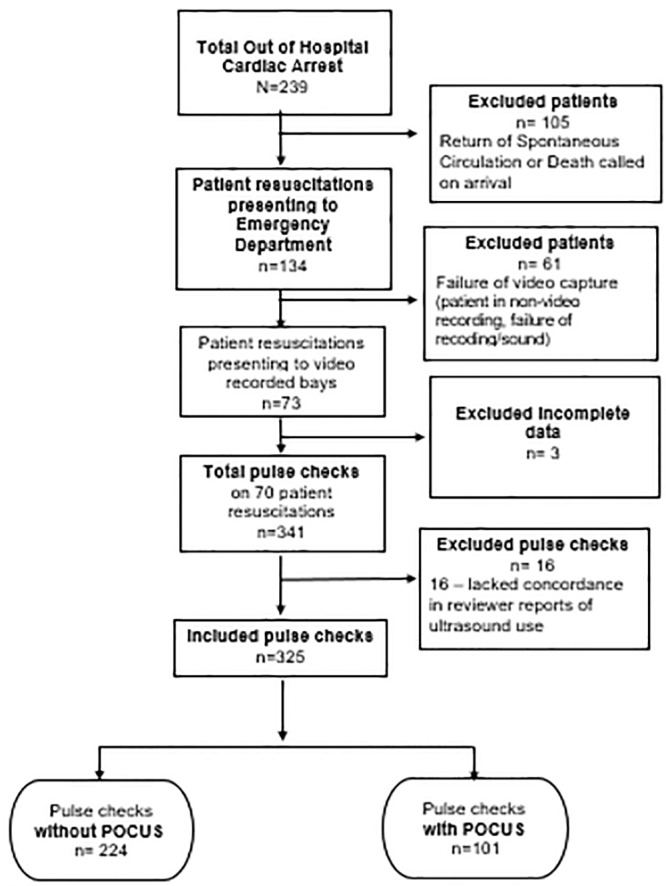
Flow diagram for patients approached for enrolment in this study. *POCUS*, point-of-care ultrasound.

Over 19 months, investigators reviewed 70 patient resuscitations. Mean age of the patients was 58.6 years old with standard deviation of 13.2. Twenty (28.8%) of the patients were female; 18 (25.7%) patients had ROSC; and three (4.3%) survived to hospital discharge (Table 1). A total of 239 patients presented to the ED in OHCA. Of those, 105 were excluded due to ROSC or death on arrival, leaving 134 eligible patients. Of the remaining patients, 61 were placed in non-videorecording rooms, or had failure of the video capture system. Three resuscitations were excluded due to incomplete data, resulting in 70 patients for analysis. (Figure 1). A total of 341 pulse checks were reviewed from the 70 patients. Sixteen pulse checks did not have concordance in reviewer reports of ultrasound use and were thus excluded, leaving 325 pulse checks for analysis (Figure 1). Interrater reliability of pulse check length was relatively strong (intraclass correlation coefficient ICC = 0.9343, r = 0.9330; *P*<0.0001). 13

**Figure 2:**
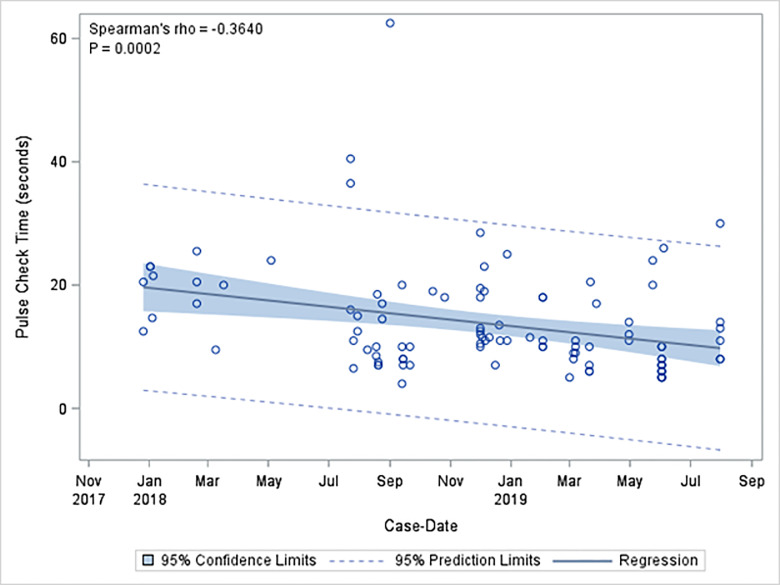
Correlation between pulse check length with ultrasound use throughout time of educational intervention.

**Figure 3:**
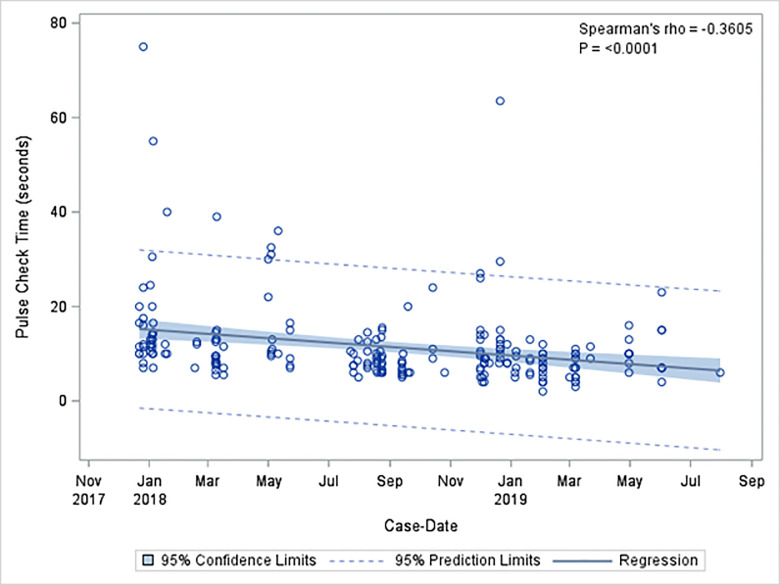
Correlation between pulse check length without ultrasound use throughout time of educational intervention.

There were 224 pulse checks without ultrasound (68.9%), and 101 pulse checks with ultrasound (31.1%). Mean length of pulse checks was 12.2 seconds with standard deviation (SD) of 8.8 seconds. The mean length of pulse checks without ultrasound was 11.5 seconds with SD of 8.8 seconds. The mean length of pulse checks that used ultrasound was 13.8 seconds with SD of 8.6 seconds. Pulse checks using ultrasound were significantly longer than those without ultrasound (*P* = 0.001). Mean pulse check duration per three-month block had a statistically significant decrease from study onset to the final study period. Mean pulse check duration divided quarterly decreased from 17.2 ± 12.2 to 10 ± 6.5 seconds (ρ = −0.2920, *P* = <0.0001] overall; pulse checks without POCUS decreased from 16.6 ± 13.2 to 10.5 ± 6.5 seconds (ρ = −0.3547, *P* = <0.0001); and pulse checks with POCUS from 19.8 vs ± 4.2 seconds to 9.88.0 seconds ± 6.6 (ρ = −0.3981, *P* <0.0001) (Table 2). Pulse check times decreased significantly over the study period of educational interventions (ρ = −0.2953, *P*<0.0001), with an even greater negative effect size in pulse check time with ultrasound use (ρ = −0.3640, *P* = 0.0002) (Figure 2). Pulse checks without ultrasound also significantly decreased over time (ρ = −0.3605, *P* = 0.0001) (Figure 3).

## DISCUSSION

The modifiers of patient outcome in CPR are limited. Reduced hands-off time through shorter pulse checks correlates with improved survival. This study demonstrated that CPR pulse check duration improved with our educational intervention and targeted feedback. With this in mind, providers may use similar methods to potentially improve patient survival by shortening pulse checks. Although all pulse checks improved over time, pulse checks using POCUS improved more than those without. Despite this, overall average of pulse check duration with POCUS was significantly longer than pulse checks without POCUS. During the last three months of study overall pulse checks were 10 seconds. To our knowledge, pulse check durations in this study were shorter than previous studies, both with and without POCUS.^17^,^21^ Through our educational intervention we were able to achieve the goal pulse check duration of 10 seconds at the end of the study period. Thus, through the implementation of an educational intervention, we improved the pulse check duration as compared to other studies.

One of the major priorities of this study was the multidisciplinary approach, targeting education and feedback at all levels of the resuscitation team (nursing, technicians, physician assistants, and physicians). Previous studies have shown improved outcomes in cardiac arrest with an integrated team approach.^27^ With integrated education at all levels of the resuscitation team, all members feel responsible for the resuscitation, not only the physician providers. Often the ultrasonographer, focused on performing the POCUS, may not pay as close attention to pulse check duration. With a multidisciplinary approach, any team member (especially team members primarily responsible for chest compression [in our setting ED technicians]) feels empowered to interrupt a POCUS to resume chest compressions; examples of this were witnessed on video review.

Video review is a low-cost, widely adopted method used for medical education.^28^ Other academic hospitals may reproduce and adopt the methods of video review described in this study to improve key parameters in CPR. Weston et al first discussed videotaping cardiac arrest cases in 1992, when their results identified poor leadership and prolonged interruption of cardiac massage as deficiencies; however, most video use remains in simulation settings due to many challenges to recording within clinical settings.^29^–^31^ These challenges include the legality of videorecording patient care, patient privacy laws, and provider litigation. This study is innovative in its utilization of video review feedback to improve POCUS in cardiac arrest.

Another strategy to help minimize interruptions in CPR is the development of POCUS protocols. 32,33 These protocols alone did not reduce pulse check duration but could be used with video feedback review to further improve CPR quality. Another advancement in the use of POCUS has been the use of trans-esophageal echocardiography (TEE) as it does not interfere with compressions in the same way transthoracic ultrasound does. Recent studies have shown that emergency physicians can perform TEE to guide resuscitation; however, it is far from widespread adoption due to the need for specialized training and equipment. 34

We demonstrated that focused individual feedback as well as conference case review decreased the duration of pulse checks. Across the entire study period, the mean length of POCUS-assisted pulse checks in our study was 13.8 seconds, and 11.8 seconds without the use of POCUS. Averaged by quarter, mean pulse check duration significantly decreased from study onset to the final study period with the final three-month period showing a pulse check duration without POCUS of 10.5 seconds, and with POCUS of 9.8 seconds. This demonstrates that the hesitancy to use ultrasound in pulse checks created by prior research should be taken with caution, as with proper education and protocols in place it is still possible to deliver high quality CPR. We encourage emergency physicians to integrate POCUS into CPR with a continuous quality improvement process to improve the metrics of cardiac arrest resuscitation, and ultimately to improve patient outcomes.

## LIMITATIONS

The major limitations of this study include the cohort design, single-center sample size, sample bias, sonographer experience, and the use of mechanical compression devices. Our study had a relatively small sample size from only one hospital; however, to our knowledge this is the largest study in the EM literature addressing duration of pulse checks with POCUS during CPR. Although 18 patients survived to hospital admission, only three patients survived to hospital discharge; this sample size is too small to draw any meaningful conclusions about the effect of this intervention on mortality. Additionally, this was a convenience sample of patients placed into videorecorded resuscitation bays. We did not account for patients with OHCA who were placed in non-video rooms as we would not have been able to extract the same data from these cases. For example, nursing staff do not record pulse check times during a typical non-video resuscitation.

The significant improvement in pulse check duration when using POCUS demonstrated during this study may simply be correlation, related to some other factor other than the educational feedback, and not causation, as there was no comparison group not receiving the feedback. For example, providers may have experienced the Hawthorne effect, and may have aimed to improve pulse check times because they were aware of being videorecorded. This probable Hawthorne effect, or the fact that focusing attention on pulse check duration during CPR impacts CCF, is in some ways not a limitation, as it informs the practice of performance assessment during resuscitations to improve the quality of CPR.

The generalizability of this intervention presents a further limitation. With the decrease in financial cost of video review technology we anticipate that other institutions may adopt this protocol to improve their practice; however, it is certainly time and resource intensive. These interventions at our hospital have continued after the study period ended, but time will tell if this proves sustainable. An additional limitation is that the experience of the sonographer obtaining cardiac views during arrest is a major factor in the length of the pulse checks.^34^ However, we did not account for the sonographers’ level of experience (eg, year of training; fellow or attending status; previous POCUS training) in our study. Therefore, we cannot further stratify pulse check lengths with the sonographer’s experience.

Finally, our hospital and EMS system frequently used a mechanical chest compression device for continuous compressions (LUCAS, Stryker Medical, Portage, MI). In this study, 65 patients (92.9%) received compressions via LUCAS device. Due to the size and placement of the LUCAS, it impairs the use of the parasternal window, a useful view in cardiac arrest when gastric distension limits the subxiphoid view. Although in practice the most commonly obtained window is the subxiphoid view, we were not able to document the view used during each ultrasound check.

## CONCLUSION

We have demonstrated that a targeted educational intervention improved pulse check times overall, and improved pulse checks with POCUS to an even greater degree. We anticipate that with further attention and intervention related to this important topic, we will continue to improve pulse check times both with and without POCUS. As our intervention is ongoing, our observation has led us to continue our study as we hope to further use this method to optimize cardiac resuscitations and minimize potential harm to our patients.

## Figures and Tables

**Table 1:** Patient characteristics by case, n=70.

Variable	Overall summary statistics	No ultrasound used	Ultrasound used
Age	58.6 ± 13.2	60.6 ± 13.9	57.8 ± 12.9
	56.5 (51, 68)	59.5 (55, 67)	56 (49, 68)
Sex			
Male	50 (71.4%)	10 (45.5%)	40 (83.3%)
Female	20 (28.6%)	12 (54.5%)	8 (16.7%)
Race			
AA/Black	45 (64.3%)	14 (63.6%)	31 (64.6%)
Hispanic	2 (2.9%)	1 (4.5%)	1 (2.1%)
White	20 (28.6%)	6 (27.3%)	14 (29.2%)
N/A	3 (4.3%)	1 (4.5%)	2 (4.2%)
Prehospital rhythm			
PEA	24 (34.3%)	8 (36.4%)	16 (33.3%)
Asystole	23 (32.9%)	6 (27.3%)	17 (35.4%)
V fib	15 (21.4%)	4 (18.2%)	11 (22.9%)
V tach	3 (4.3%)	1 (4.5%)	2 (4.2%)
Unknown	5 (7.1%)	3 (13.6%)	2 (4.2%)
Compression device			
Hands	5 (7.1%)	2 (9.1%)	3 (6.3%)
Lucas	65 (92.9%)	20 (90.9%)	45 (93.7%)
Ultrasound was used at somepoint	48 (68.6%)	-	48 (100%)
ER outcome			
Admitted to hospital	18 (25.7%)	10 (45.5%)	8 (16.7%)
Death	52 (74.3%)	12 (54.5%)	40 (83.3%)
Survived hospital discharge	3 (4.3%)	3 (13.6%)	-

Reported as # (%), mean ± standard deviation, and/or median (interquartile range).

*AA*, African American; *PEA*, pulseless electrical activity; *ER*, emergency room; *V fib*, ventricular fibrillation; *V tach*, ventricular tachycardia.

**Table 2:** Pulse check length by every three months of study time (QUARTERLY).

Sample	Mean ± SD	Spearman’s ρ	P-value
Overall (n=325)	12.2 ± 8.8	−0.2953	<0.0001
Q1	17.2 ± 12.0	−0.2920	<0.0001
Q2	14.0 ± 8.8		
Q3	10.8 ± 6.5		
Q4	11.6 ± 11.3		
Q5	11.5 ± 7.9		
Q6	9.7 ± 4.6		
Q7	10.0 ± 6.5		
Without US (n=224)	11.5 ± 8.8	−0.3605	<0.0001
Q1	16.6 ± 13.2	−0.3547	<0.0001
Q2	13.6 ± 8.9		
Q3	9.1 ± 2.8		
Q4	8.9 ± 5.2		
Q5	10.3 ± 8.5		
Q6	8.3 ± 3.1		
Q7	10.5 ± 6.5		
With US (n=101)	13.8 ± 8.6	−0.3640	0.0002
Q1	19.8 ± 4.2	−0.3981	<0.0001
Q2	17.8 ± 7.5		
Q3	14.8 ± 10.0		
Q4	15.8 ± 16.4		
Q5	14.4 ± 5.4		
Q6	11.7 ± 5.6		
Q7	9.8 ± 6.6		

*US*, ultrasound; *SD*, standard deviation.
